# Mechanical Properties Testing and Numerical Modeling and Simulations of a Nozzle Cover Made of Expanded Polystyrene

**DOI:** 10.3390/ma18163835

**Published:** 2025-08-15

**Authors:** Jianyong Jiang, Zhixuan Zhang, Jian Zheng, Kehui Shu, Wenhao Zhu

**Affiliations:** 1 Key Laboratory of Special Engine Technology, Ministry of Education, School of Mechanical Engineering, Nanjing University of Science and Technology, Nanjing 210094, China; susu556692025@163.com (J.J.); 15122173259@163.com (Z.Z.); a1339010@163.com (W.Z.); 2 Jiangnan Industrial Group Co., Ltd., Xiangtan 411100, China; 13667419950@163.com

**Keywords:** expandable polystyrene (EPS), nozzle cover, mechanical properties, constitutive model, numerical simulation, VUMAT subroutine

## Abstract

Expandable polystyrene (EPS) nozzle covers can be used to replace traditional metal nozzle covers due to their excellent mechanical properties, as well as being lightweight and ablatable. As an important part of the solid rocket motor, the nozzle cover needs to be designed according to the requirements of the overall system. This study lays a theoretical foundation for the engineering design and performance optimization of the EPS nozzle cover. In this paper, the method of combining test research and numerical simulation is used to explore the pressure bearing capacity of EPS nozzle covers with different thicknesses under linear load. Firstly, the quasi-static tensile, compression and shear tests of EPS materials were carried out by universal testing machine, and the key parameters such as stress-strain curve, elastic modulus and yield strength were obtained; Based on the experimental data, the constitutive model of EPS material with respect to density is fitted and modified; The VUMAT subroutine of the material was written in Fortran language, and the mechanical properties of the nozzle cover with different material model distribution schemes and different thicknesses were explored by ABAQUS finite element numerical simulation technology. The results indicate that the EPS nozzle cover design based on the two material model allocation schemes better aligns with practical conditions; when the end thickness of the EPS nozzle cover exceeds 3 mm, the opening pressure formula for the cover based on the pure shear theory of thin-walled circular plates becomes inapplicable; the EPS nozzle cover exhibits excellent pressure-bearing capacity and performance, with its pressure-bearing capacity showing a positive correlation with its end thickness, and an EPS nozzle cover with a 9 mm end thickness can withstand a pressure of 7.58 MPa (under internal pressure conditions); the pressure-bearing capacity of the EPS nozzle cover under internal pressure conditions is higher than under external pressure conditions, and when the end pressure-bearing surface thickness increases to 9 mm, the internal pressure-bearing capacity is 3.13 MPa higher than under external pressure conditions.

## 1. Introduction

As an important part of the solid rocket motor, the main functions of the nozzle cover are as follows: before ignition, it plays an important role in sealing moisture proof and maintaining internal pressure balance, avoiding the moisture of the internal grain and grain box of the motor, and ensuring the normal operation of the propellant and ignition powder; during ignition, the ignition pressure of the engine is established, the ignition delay period is shortened, and the ignition reliability is improved. In the design, the material selection, thickness, placement position and failure pressure should be studied in depth [1].

The nozzle cover can be divided into a metal-based nozzle cover and a non-metal nozzle cover according to its substrate properties. The research on metal-based nozzle cover mainly includes: Chen Changjiang et al. used 1Cr18Ni9Ti stainless steel. The single-layer and double-layer structures were compared by experiment and simulation. It was found that the double-layer design significantly improved the pressure bearing capacity [2]; fan Ding et al. used aluminum alloy material to optimize the structural design by combining test and simulation, which effectively improved the opening reliability of the nozzle cover [3]. In addition, Liu Daokun et al. developed a metal-based composite nozzle cover, which uses aluminum alloy as the substrate to composite WL-640 insulation layer, and verified its excellent thermal insulation performance under cold flow conditions through experiments and simulations [4]. The study of non-metallic nozzle cover includes: Tu Xiaochang et al. prepared a single material polyurethane rigid foam nozzle cover, and the test confirmed its feasibility [5]; zhang Meng et al. developed a composite non-metallic nozzle cover, using rubber and sandwich composite materials, and innovatively applied a simplified finite element model for simulation analysis (error as low as 5.3%), which provides a reliable simulation method support for the design of nozzle cover of this kind of material [6].

In summary, most of the existing studies on nozzle cover are based on conventional materials with known material parameters. Although some literature [6] explored new materials, their research only focuses on the specific material itself, failing to cover the performance changes under different densities, and it is difficult to provide a basis for subsequent scholars to design such material products similar to the parameterized design of metal materials.

Conventional solid rocket motors mostly use metal-based nozzle covers, because the metal material is difficult to ablate or can not be completely ablated, high-speed flying out of the nozzle cover debris may pose a serious threat to the rear personnel and the launch platform. Therefore, there is an urgent need to develop a new type of nozzle cover material—not only to maintain the mechanical properties of metal materials, but also to be able to quickly and completely ablate after engine ignition, thus fundamentally eliminating this safety risk.

With the development of materials science and engineering, many non-metallic materials now possess mechanical properties comparable to those of metallic materials, while also exhibiting numerous characteristics that metallic materials lack.

Polymer foam, as a typical non-metallic material, mainly contains three kinds of foamed plastics, such as polystyrene (EPS), polypropylene (EPP) and polyethylene (EPE). The EPS material is characterized by light weight, high flexural modulus, high multiplicity, and an easy-to-produce closed-cell structure [7]. As a material with excellent performance, EPS can not only be used alone, but also can be further optimized by compounding with other materials. According to its combination, it can be divided into stacked combination type optimization and matrix material hybrid optimization. In the study of stacked combination type optimization, Zexiong Zhang et al. investigated the mechanical properties and cushioning properties of flexible polyurethane foam (FPUF) and EPS combination materials with different structural scale parameters through theoretical analysis and finite element simulation, revealing the complementary advantages of parallel and tandem structures [8]. Itsara Rojana et al. tested aluminum honeycomb structures partially filled with EPS foams by axial quasi-static compression tests. Tests on an aluminum honeycomb structure partially filled with EPS foam found that a specific amount of partial filling can significantly enhance its energy absorption capacity and mean crush force [9]. Of the matrix material mix optimization, Quan You et al. investigated the fatigue and durability behavior of structural EPS concrete, and the results showed that, compared with ordinary concrete of the same strength, the material has superior energy absorption capacity and toughness, and exhibits excellent fatigue stability, and also in the wet-dry (W-D) cyclic test, the structural EPS concrete also demonstrated good sulfate corrosion resistance [10].

EPS material not only has excellent performance, but also has excellent processing adaptability. The material can be processed into different shapes and sizes by cutting, molding [11] and other molding processes, which can flexibly meet the customization needs of various application scenarios.

It can be seen that the use of EPS material to make a nozzle cover has the following advantages: lightweight; impact resistance; heat insulation; corrosion resistance; easy to mold; low cost; no residue of burnt fusion.

With the development of finite element analysis technology, the use of finite element software to analyze the nozzle cover has become an indispensable key link in the design and analysis of the nozzle cover. Numerical simulation replaces some of the expensive tests and significantly reduces R&D costs. In Reference [12], ABAQUS finite element simulation software was used to analyze the influence of the opening pressure of the cover, the pressure building rate of the combustion chamber and the density of the cover material on the opening process of the cover.

This paper employs a combined experimental, theoretical, and numerical simulation approach to systematically investigate the pressure-bearing capacity of EPS nozzle covers. First, tensile, compressive, and shear mechanical property tests were conducted to obtain stress-strain data for EPS samples with densities ranging from 0.13 to 0.58 g/cm^3^ (10 uniformly spaced density groups), and the influence of density on mechanical properties was analyzed. Based on the test data, a constitutive model was established to characterize the mechanical behavior of EPS material as a function of density. The model was modified to significantly improve its fitting accuracy to the stress-strain test data. Subsequently, based on the modified and optimized constitutive model, a VUMAT user subroutine for EPS material was developed, and the model was validated using ABAQUS 2023 finite element analysis software and its user subroutine interface. Based on the primary stress characteristics of the nozzle cover under actual operating conditions, it was divided into several characteristic regions, and each region was assigned verified material properties. Through preliminary simulation verification, the three-dimensional finite element model was determined. Finally, using this model, a systematic study was conducted on the stress distribution patterns and changes in pressure-bearing capacity of nozzle cover structures with different thicknesses under the action of internal and external pressure loads.

This study provides theoretical support for the structure optimization and performance design of the solid rocket motor EPS nozzle cover and other EPS products.

## 2. Materials and Methods

### 2.1. Specimen Preparation

The test material is Longwang brand E-S grade EPS produced by Dongguan Xinchangqiao Plastic Co., Ltd. (Dongguan, China).

**Figure 1:**
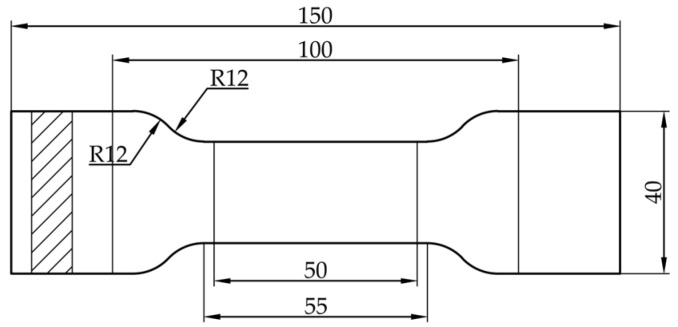
Tensile specimen size.

The ISO 1926-2009 standard [13] specifies the shape and dimensions of EPS tensile test specimens (Figure 1), the size unit in the figure is mm.

**Figure 2:**
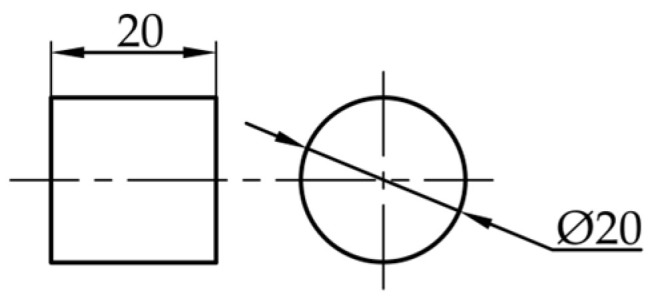
Compression sample size.

According to the maximum range limit of the universal material testing machine, the shape and size of the compressed sample are designed as a cylinder with a diameter of 20 mm and a height of 20 mm (Figure 2).

**Figure 3:**
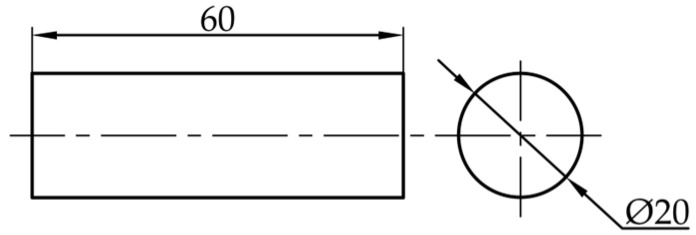
Shear specimen size.

The shape and size of the shear specimen are designed as a cylinder with a diameter of 20 mm and a height of 60 mm (Figure 3).

**Figure 4:**
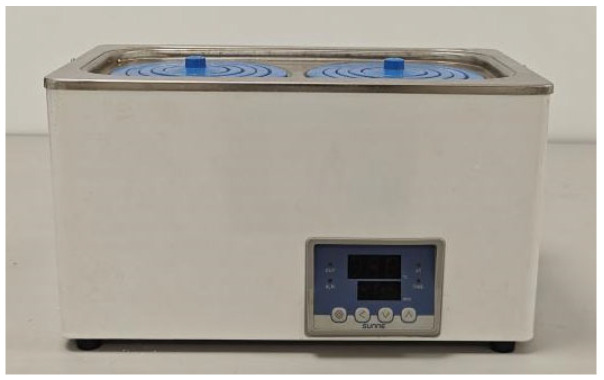
Anti-dry boiled water bath.

Customized molds were made based on the sample dimensions, and an SN-HWS-2F anti-dry burning water bath pot (Figure 4) manufactured by Shanghai Shangpu Instrument Equipment Co., Ltd. (Shanghai, China) was used to prepare ESP test pieces of different densities by water bath foaming.

### 2.2. Test Equipment and Test Methods

The test equipment is a QJ-211B test machine manufactured by Shanghai Qingji Instrumentation Technology Co., Ltd. (Shanghai, China).

Test methods: Tensile test refers to ISO 1926-2009 standard [13], compression test refers to ISO 844-2014 standard [14], and shear test refers to ISO 1922-2018 standard [15].

**Figure 5:**
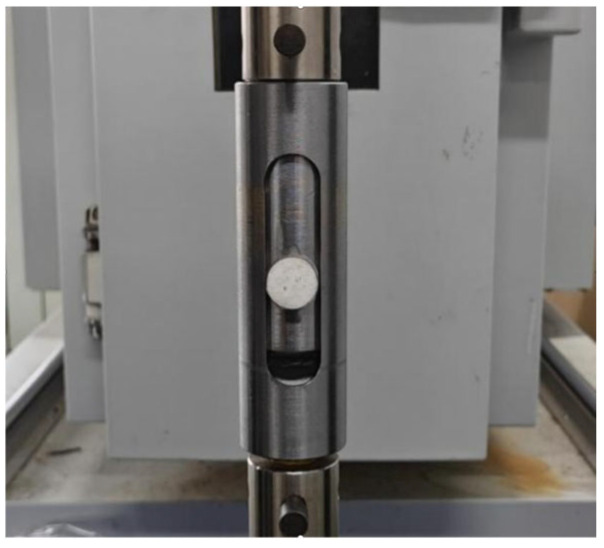
Shear test fixture.

The test fixture shown in Figure 5 was designed based on the shape and dimensions of the shear test specimens.

## 3. Test Results and Analysis

**Figure 6:**
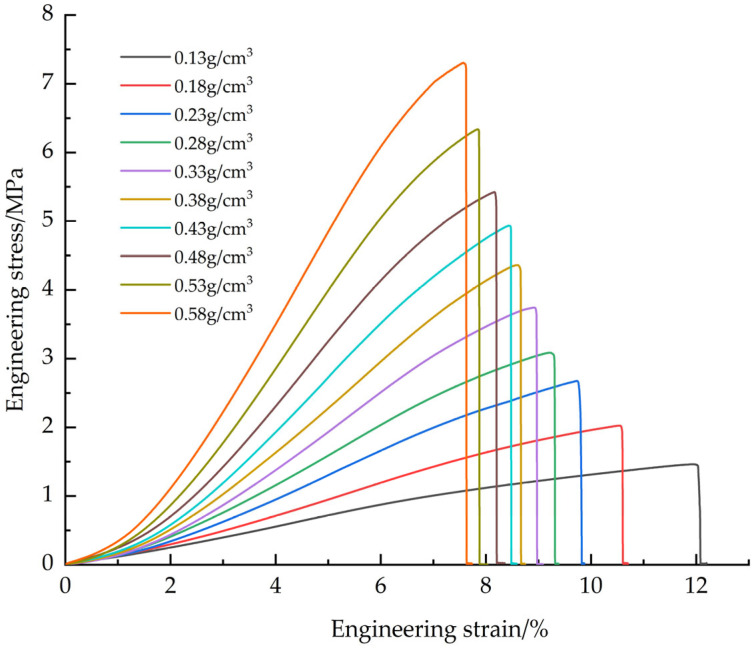
Average engineering stress-strain curve of tensile test.

**Figure 7:**
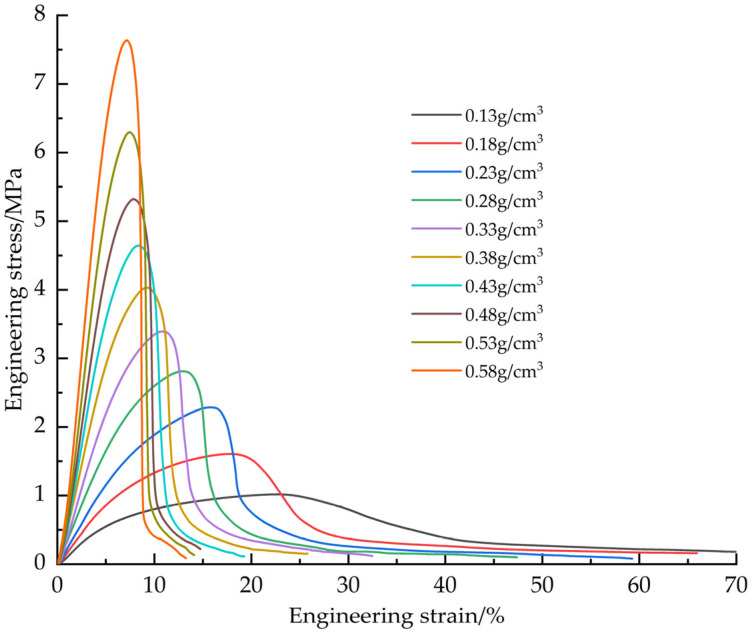
Average engineering stress-strain curve of shear test.

**Figure 8:**
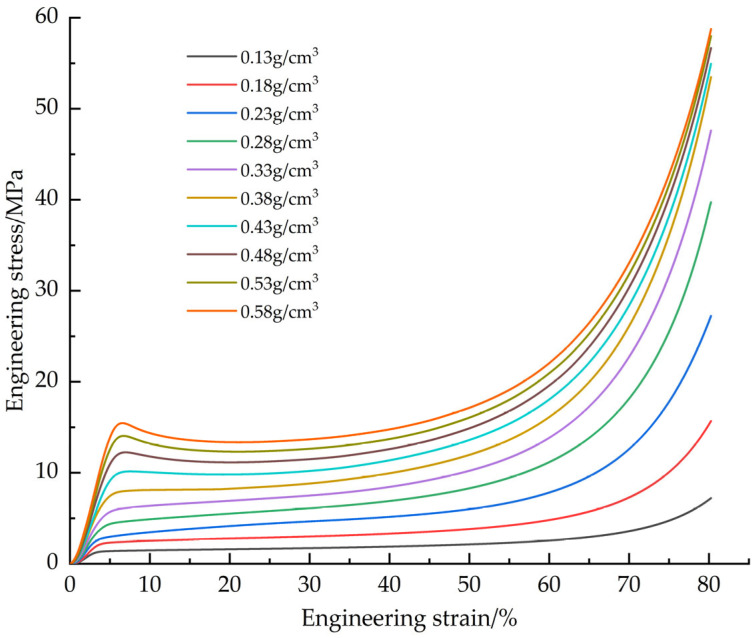
Average engineering stress-strain curve of compression test.

The EPS samples with densities of 0.13, 0.18, 0.23, 0.28, 0.33, 0.38, 0.43, 0.48, 0.53 and 0.58 g/cm^3^ were tested. The tensile average engineering stress-strain curves of EPS with different densities (Figure 6), EPS shear average engineering stress-strain curves (Figure 7) and compression average engineering stress-strain curves (Figure 8) were obtained.

Young’s modulus is determined by the slope of the initial linear segment of the engineering stress-strain curve. Analysis of the stress-strain curves for tensile and shear tests showed that the point of maximum stress corresponded to the failure strain of the material. For compression tests, although the stress-strain curves do not show obvious failure characteristics, the turning point at the transition from the elastic phase to the plastic plateau can be used as the yield point. The data obtained based on tensile, shear and compression tests are presented in Table 1, Table 2 and Table 3: Young’s modulus (all three tables), as well as maximum stress and failure strain (Table 1 and Table 2), yield stress and yield strain (Table 3).

The failure modes of EPS material in tensile and shear tests are mainly crack propagation and hole wall fracture between foam walls, and crack propagation gradually dominates with the increase of density. In the compression test, the failure mode is the crushing of foam beads. Due to the volume limitation, increasing the density of the material requires filling more EPS beads in the same volume, resulting in a decrease in the degree of foaming of a single bead and a decrease in the proportion of foam, thereby increasing the hardness of the material. The mechanical properties and density of materials show regular changes: Young‘s modulus, failure stress and yield stress increase with the increase of density. In tensile and shear tests, the failure strain decreases with increasing density. In the compression test, the yield strain increases with the increase of density and finally tends to be stable (about 6.5%). At the same time, with the increase of density, the platform stage of the engineering stress-strain curve is shortened, and the densification stage appears in advance. When the density exceeds 0.33 g/cm^3^, there will be a stress drop section in the critical region of the transition from the elastic stage to the platform stage, and this phenomenon becomes more significant with the increase of density. The above density-related regular changes provide a good basis for the establishment of the EPS variable density constitutive model.

## 4. Construct EPS Constitutive Model

### 4.1. EPS Tensile Constitutive Model

The tensile constitutive model of EPS was adopted from the Sherwood-Frost model, which is a temperature, strain, density and strain rate separable constitutive model of foam materials obtained by Sherwood and Frost [16] after summarization:

(1) $$\sigma =H\left(T\right)G\left(\rho \right)M\left(\varepsilon ,\dot{\varepsilon }\right)f\left(\varepsilon \right)$$

Combined with the fact that only the effect of material density is considered in this paper, and the influencing factors of temperature and strain rate are not considered for the time being, the constitutive equation is obtained as:

(2) $$\sigma =G\left(\rho \right)f\left(\varepsilon \right)$$

#### 4.1.1. Model Parameter Identification

The shape functions are polynomial expressions proposed by Schwaber and Meinecke et al. [17] in 1971.

(3) $$f\left(\varepsilon \right)=\sum_{i=1}^{n}A_{i}\varepsilon^{i}$$

In the formula, $A_{i}$ a is the fitting parameter.

**Figure 9:**
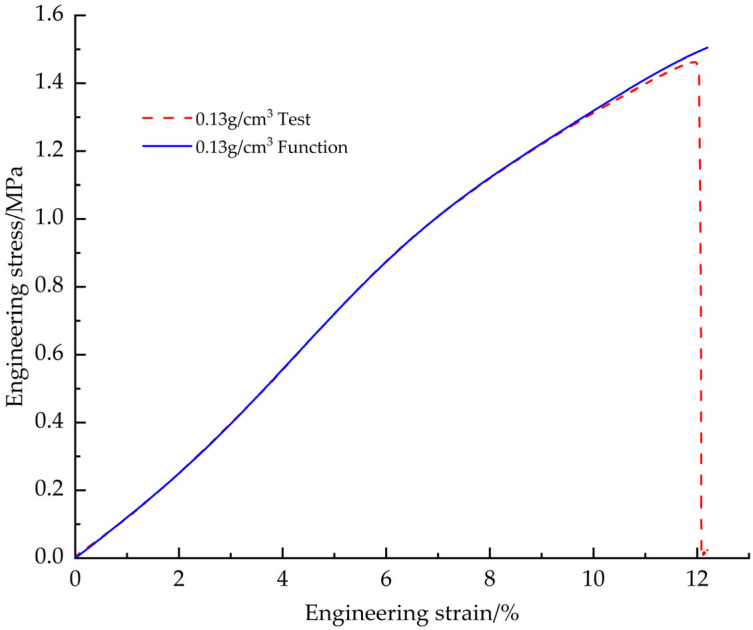
EPS tensile constitutive model shape function fitting curve vs. test curve.

The fitting results based on the shape function model showed that the best fitting results were obtained for the test data with a density of 0.13 g/cm^3^ when the shape parameter n = 8 (Figure 9). The specific values of the fitting parameters are shown in detail in Table 4.

In the Sherwood-Frost constitutive model, it is considered that the effect of density on stress is single. Hu et al. [18] studied the constitutive relationship of rigid polyurethane foam. Through the experimental results, it was found that the effect of density on stress was also related to the strain; that is, the strain and density were coupled to affect the stress. Therefore, the density term in the Sherwood-Frost constitutive model is changed into a term $G\left(\frac{\rho }{\rho_{0}},\varepsilon \right)$ containing strain, which is expressed in a power exponential form.

(4) $$G\left(\frac{\rho }{\rho_{0}},\varepsilon \right)={\left(\frac{\rho }{\rho_{0}}\right)}^{A}\cdot e^{B\left(\frac{\rho }{\rho_{0}}-1\right)\varepsilon }$$

In the formula: A and B are the fitting parameters.

With 0.13 g/cm^3^ as the reference density, the unknown parameters A and B of the density term were determined by fitting. Table 5 lists the parameters corresponding to the other nine densities.

The results show that neither parameter A nor B presents distribution characteristics around a fixed value, so it is not appropriate to use the average value for characterization. Further analysis reveals that the variation rule of parameter B is highly similar to the tensile fracture engineering strain, and the two show significant linear correlation.

(5) $$B\left(e_{r}\right)=0.01697e_{r}-0.117$$

In the formula, $e_{r}$ is the engineering strain at tensile fracture.

According to the above test results, the relationship between engineering strain and density during tensile fracture of EPS can be established as follows:

(6) $$e_{r}\left(\rho \right)=6.638\times \rho^{-0.2645}$$

Therefore, the parameter B is related to the density of EPS as:

(7) $$B\left(\rho \right)=0.11264686/\rho^{0.2645}-0.117$$

Updating the fitted value of parameter A according to this functional relationship, the relationship between parameter A and EPS density can be obtained as:

(8) $$A\left(\rho \right)=1.243\rho +0.3477$$

#### 4.1.2. Model Validation and Correction

**Figure 10:**
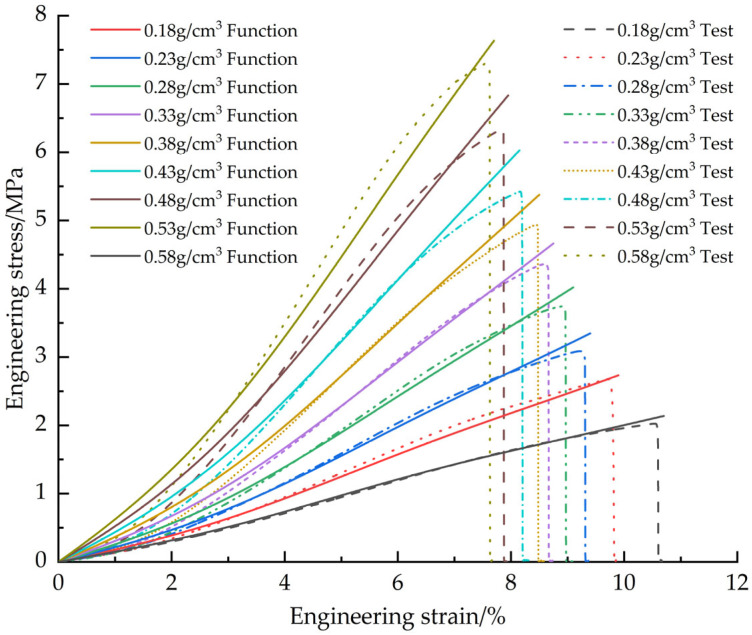
Original EPS tensile constitutive model curves vs. test curves.

Figure 10 shows the fitted curves of the EPS tensile constitutive model based on the Sherwood-Frost model with the test curve

The results show that the current constitutive model is poorly fitted in the initial stage of tensile and near-fracture regions. In order to more accurately characterize the stress-strain response of EPS materials during the entire tensile test, it is necessary to introduce an additional correction term into the existing constitutive equation. It is found that the addition of the correction term $a\varepsilon^{b}e^{c\varepsilon }$ can greatly improve the fit of the fitted curve. The correction term a controls the overall magnitude of the curve, $\varepsilon^{b}$ controls the growth or decay trend of the curve at small strains, and $e^{c\varepsilon }$ controls the growth or decay trend at large strains. The corrected EPS constitutive equation is:

(9) $$\sigma =\sum_{i=1}^{n}A_{i}\varepsilon^{i}\cdot {\left(\frac{\rho }{\rho_{0}}\right)}^{A}\cdot e^{B\left(\frac{\rho }{\rho_{0}}-1\right)\varepsilon }a\varepsilon^{b}e^{c\varepsilon }$$

The parameters a, b, c are related to the EPS density as:

(10) $$a\left(\rho \right)=0.6827+0.02249\cos\left(8.189\rho \right)+0.09656\sin\left(8.189\rho \right)$$

(11) $$b\left(\rho \right)=1.817\rho -0.06844$$

(12) $$c\left(\rho \right)=-0.3936\rho +0.0353$$

**Figure 11:**
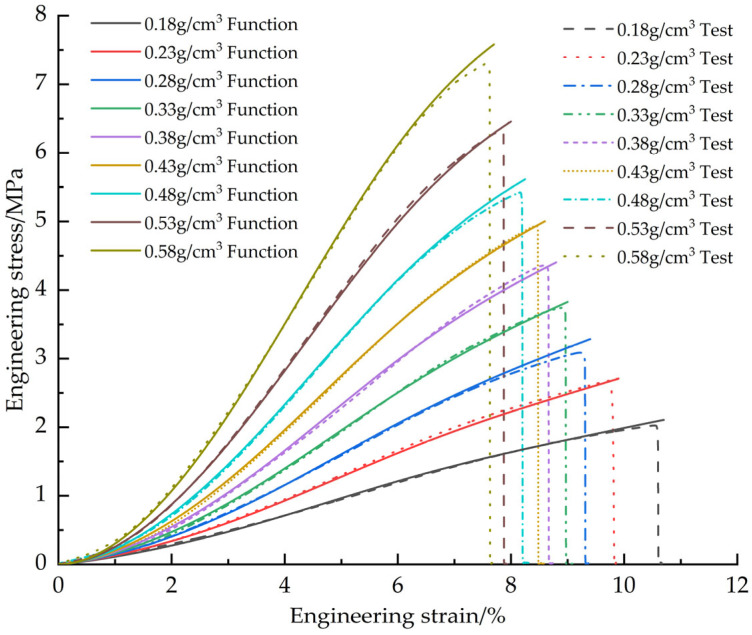
Corrected EPS tensile constitutive model curves vs. test curves.

Figure 11 illustrates the corrected EPS tensile constitutive fitting curves and test curves.

**Figure 12:**
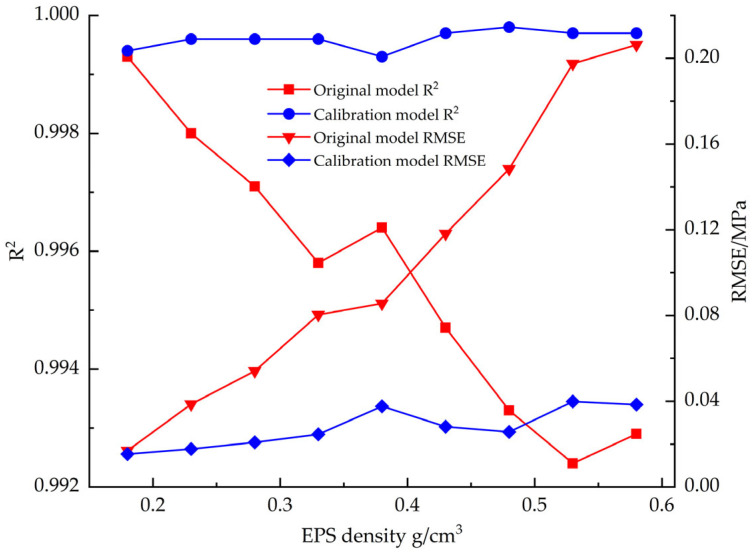
R^2^ vs. RMSE before and after EPS tensile constitutive model correction.

Figure 12 shows the changes in goodness-of-fit (R^2^) and root mean square error (RMSE) between the EPS tensile constitutive model and the test data before and after correction. The corrected constitutive model shows excellent fitting performance: the mean value of R^2^ is improved to 0.9996 compared to 0.9955 before correction, while the mean value of RMSE is decreased to 0.0276 compared to 0.1051 before correction.

**Figure 13:**
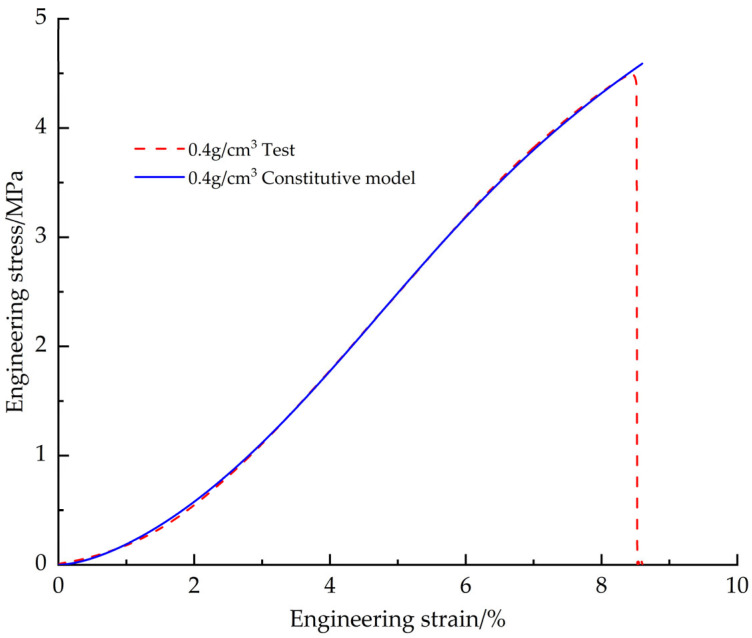
Corrected EPS tensile constitutive model curve vs. test curve.

In order to verify the accuracy of the corrected EPS tensile constitutive model, the EPS tensile test data with a density of 0.4 g/cm^3^ were used for comparison (Figure 13). The results show that the engineering stress-strain curve obtained from the test fits the predicted curve of the constitutive model with a goodness of fit of 0.9998 and a root mean square error of 0.0156, which indicates that the modified EPS tensile constitutive model is able to accurately predict the tensile mechanical behavior of the EPS materials.

### 4.2. EPS Shear Constitutive Model

Although the tensile test shows necking followed by fracture, while the shear test shows shear band formation, slip until final failure, they are very different failure phenomena; the fundamental reason is that the microscopic mechanisms are completely different. However, based on the similarity between the EPS shear stress-strain curve and the tensile stress-strain curve—i.e., both of them show a slow rise, followed by a sharp rise, and then a slow rise, and the maximum stress point reached is the failure strain point at the same time, the shear constitutive model of EPS is also based on the Sherwood-Frost model, which is the same as the Sherwood-Frost model. Sherwood-Frost model. Meanwhile, given that only the effect of material density is considered in this paper, the shear constitutive model is formally consistent with the unmodified tensile constitutive model of EPS.

#### 4.2.1. Model Parameter Identification

**Figure 14:**
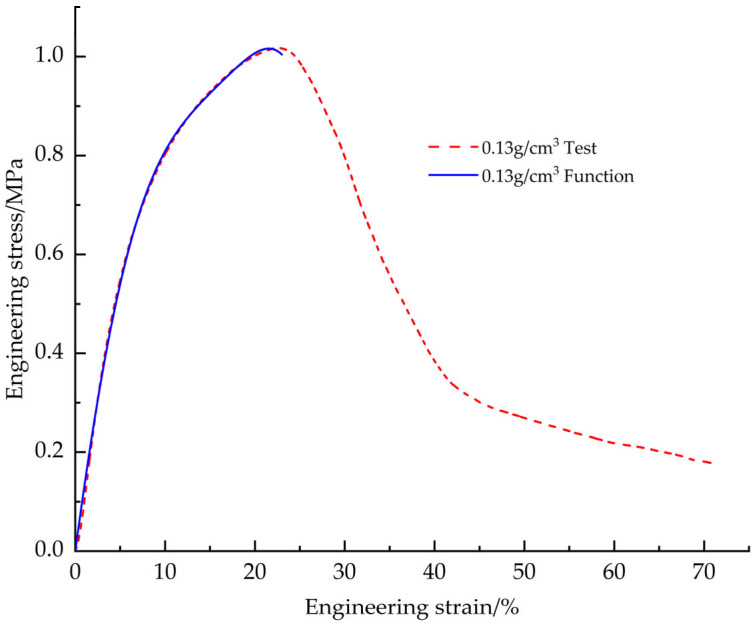
EPS shear constitutive model shape function fitting curve vs. test curve.

The EPS test stress-strain data with a density of 0.13 g/cm^3^ was fitted using a shape function and found to have a good fit at n = 5. Figure 14 demonstrates the fit curve to the test curve, and Table 6 shows the values of the shape parameter fitting parameters.

The density term was also adopted from the density term function rewritten by Hu et al. [18] in their study of the constitutive relationship of rigid polyurethane foam. The fitting of the density term parameters based on the reference density of 0.13 g/cm^3^ showed that the parameters A and B were uniformly distributed around 1.432 and 0.009097. Therefore, the finalized values of the parameters were determined to be A = 1.432 and B = 0.009097.

#### 4.2.2. Model Validation and Correction

**Figure 15:**
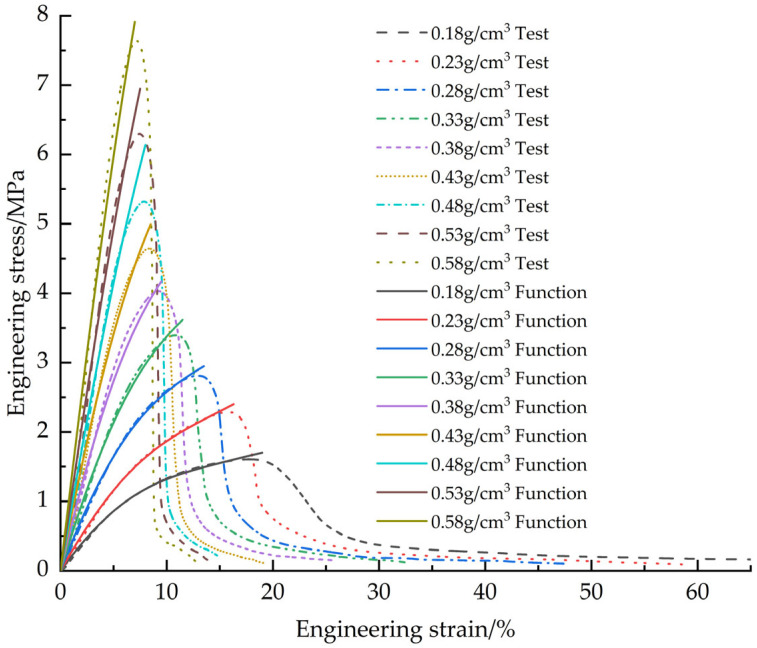
Original EPS shear constitutive model curves vs. test curves.

Figure 15 shows the fitted curves of the EPS shear constitutive model based on the Sherwood-Frost model with the test curve.

From Figure 15, it can be seen that the reason for the poor fit of the EPS shear constitutive model based on the Sherwood-Frost model is similar to the case of the uncorrected EPS tensile constitutive model, so the same correction term can be used for optimization, and in view of the fact that the shear stress-strain curve has a sharp increase in the stress at smaller strains, $a\varepsilon^{b}$ cannot satisfy the demand, and the optimization rewritten as $a{\left(1+\varepsilon \right)}^{b}$, the fit can be greatly improved, the correction term added to the shear constitutive model is $a{\left(1+\varepsilon \right)}^{b}e^{c\varepsilon }$. The corrected EPS shear constitutive model equation is:

(13) $$\sigma =\sum_{i=1}^{n}A_{i}\varepsilon^{i}{\left(\frac{\rho }{\rho_{0}}\right)}^{A}e^{B\left(\frac{\rho }{\rho_{0}}-1\right)\varepsilon }a{\left(1+\varepsilon \right)}^{b}e^{c\varepsilon }$$

The reference density of 0.13 g/cm^3^ was selected, and the relationship between parameters a, b, c and the density was obtained by least-squares fitting using the test data of EPS at this density as a benchmark, as follows:

(14) $$a\left(\rho \right)=0.5448-0.1976\cos\left(11.42\rho \right)+0.0934\sin\left(11.42\rho \right)$$

(15) $$b\left(\rho \right)=0.7433+0.3941\cos\left(10.07\rho \right)-0.444\sin\left(10.07\rho \right)$$

(16) $$c\left(\rho \right)=-0.1257-0.05469\cos\left(9.383\rho \right)+0.1016\sin\left(9.383\rho \right)$$

**Figure 16:**
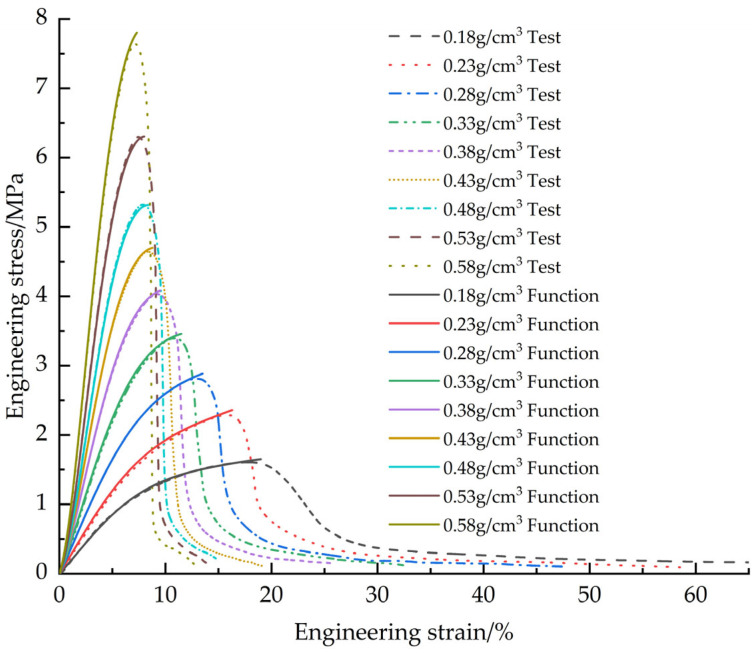
Corrected EPS shear constitutive model curves vs. test curves.

Figure 16 shows the correction of the corrected EPS shear constitutive model fit curve to the test curve.

**Figure 17:**
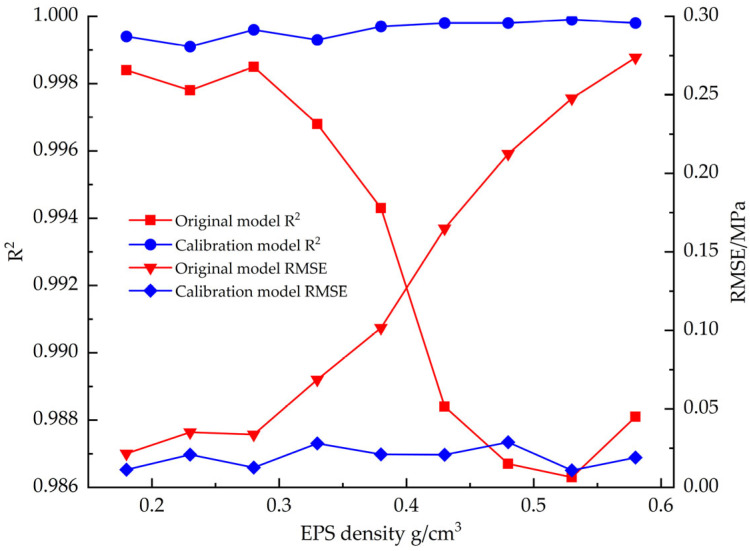
R^2^ vs. RMSE before and after EPS shear constitutive model correction.

Figure 17 demonstrates that the corrected EPS shear constitutive model has a substantially better fit than the pre-corrected constitutive model, where the mean value of R^2^ is improved from 0.9928 to 0.9996 and the mean value of RMSE is reduced from 0.1288 to 0.01925.

**Figure 18:**
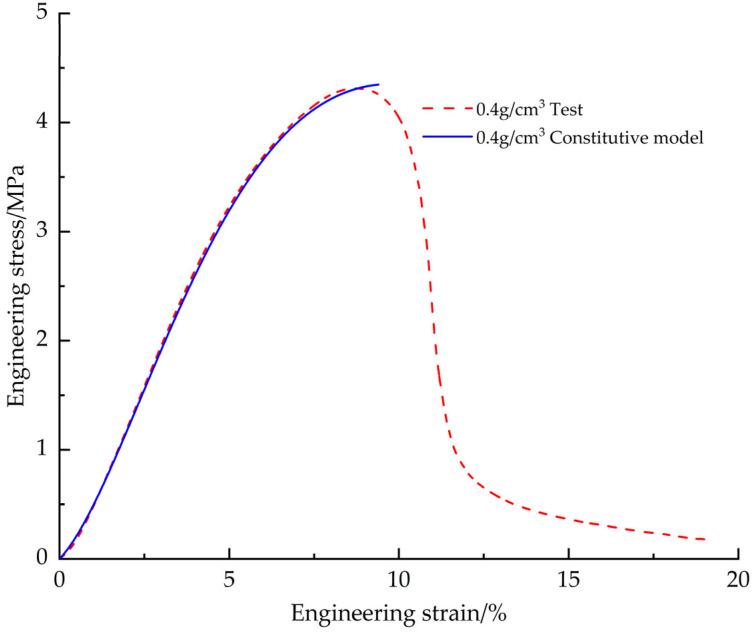
Corrected EPS shear constitutive model curve vs. test curve.

In order to verify the accuracy of the corrected EPS shear model, the EPS shear test data with a density of 0.4 g/cm^3^ were also used for comparison (Figure 18). The goodness of fit between the engineering stress-strain curve obtained from the test and the predicted curve of the model is 0.9995, and the root mean square error is 0.0314. In conclusion, the corrected EPS shear model is able to accurately predict the shear mechanical behavior of EPS materials.

### 4.3. EPS Compression Constitutive Model

In this test, the specimen was prepared by using EPS foam material, and the stress-strain curve was obtained through the foam material compression test. Therefore, the curve presents the typical compression stress-strain curve of foam material characterized by three stages: elastic deformation stage, yield plateau stage and densification stage.

In the study of compression constitutive modeling of foam materials, the existing methods mainly include three categories: one is based on the three-stage characteristics of the stress-strain curve to construct an exclusive constitutive model [19], the second is based on the Sherwood-Frost constitutive model to construct an constitutive model [20], and the third is based on the theoretical mathematical model of the Avalle foamed plastics to establish an constitutive model [21]. It is found that the method based on curve characteristics has obvious limitations in dealing with large density span data; the Sherwood-Frost model has limited adaptability to nonlinear deformation of stress-strain data; in contrast, the Avalle theoretical mathematical model of foamed plastics [22] can not only characterize the significant nonlinear mechanical response, but also its parameters have a clear physical meaning. Based on the above analysis, the Avalle mathematical model was finally selected in this paper to construct the compression constitutive model of EPS foam, whose specific expression is:

(17) $$\sigma =A\left(1-e^{\varepsilon \left(-E/A\right){\left(1-\varepsilon \right)}^{m}}\right)+B{\left(\frac{\varepsilon }{1-\varepsilon }\right)}^{n}$$

In the formula: A is the yield stress of the material, B is the strengthening coefficient of the material, E is the Young’s modulus of the elastic stage of the material, and m and n is the fitting parameter.

Expanding the strain ε in this equation by a factor of 100 yields the Avalle mathematical model as:

(18) $$\sigma =A\left(1-e^{\left(\varepsilon \left(-E/A\right){\left(100-\varepsilon \right)}^{m}\right)/100^{m+1}}\right)+B{\left(\frac{\varepsilon }{100-\varepsilon }\right)}^{n}$$

#### 4.3.1. Model Parameter Identification

Three key mechanical parameters of the material can be determined based on the test data: the yield stress is taken as the stress value corresponding to the turning point of the elastic phase and the plastic plateau in the stress-strain curve; the reinforcement coefficient is obtained by calculating the slope of the stress-strain curve at the plastic plateau stage; and Young’s modulus at the elastic phase is determined by the slope of the linear segment at the elastic deformation stage. The relationship between these three parameters (E, A and B) and the material density is given by the equation:

(19) $$E\left(\rho \right)=640\rho -34.62$$

(20) $$A\left(\rho \right)=-1.053+32.5\rho -178.4\rho^{2}+615.1\rho^{3}-551.3\rho^{4}$$

(21) $$B\left(\rho \right)=-4.937+53.96\rho -67.77\rho^{2}-77.17\rho^{3}+128\rho^{4}$$

The values of the unknown parameters m and n were obtained by fitting the test data (Table 7).

#### 4.3.2. Model Validation and Correction

**Figure 19:**
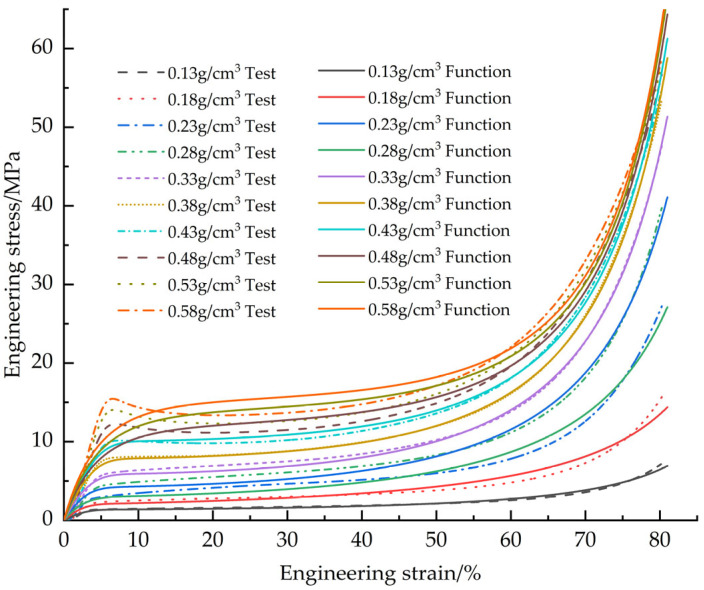
Original EPS compression constitutive model curves vs. test curves.

Figure 19 shows the fitted curves of the EPS compression constitutive model based on the Avalle mathematical model with the test curves.

It can be seen that the fitting accuracy shows a tendency to improve and then deteriorate throughout the density interval. In order to improve the model accuracy, the Avalle mathematical model needs to be optimally corrected:

First, to address the problem of insufficient fitting in the elastic interval, an adjustment coefficient c is introduced into the yield stress term; second, an adjustment coefficient d is added to the reinforcement coefficient, since the platform stage is defined from the yield point, but a falling section of the stress-strain curve occurs first after the material yields at high densities.

However, the fitting accuracy is still deficient after the introduction of the adjustment factor. It was studied further that the growth and decay characteristics of the curves could be effectively regulated by adding the correction term $a{\left(\frac{\varepsilon }{100}\right)}^{b}$ to the term containing Young’s modulus, thus improving the fitting accuracy. Eventually, the corrected EPS compression constitutive equation is:

(22) $$\sigma =cA\left(1-e^{\left(\varepsilon \left(\frac{-E}{cA}\right){\left(100-\varepsilon \right)}^{m}\right)/100^{m+1}}\right)a{\left(\frac{\varepsilon }{100}\right)}^{b}+dB{\left(\frac{\varepsilon }{100-\varepsilon }\right)}^{n}$$

The unknown parameter a, b, c, d, m, n is related to the EPS density as:

(23) $$\begin{array}{c} a\left(\rho \right)=-259.3759\rho +2652.5745\rho^{2}-13706.655\rho^{3}+39653.7979\rho^{4}\\ -65478.6933\rho^{5}+57846.2222\rho^{6}-21257.1428\rho^{7}+10.125 \end{array}$$

(24) $$b\left(\rho \right)=-0.0195-0.05621\cos\left(9.196\rho \right)+0.1772\sin\left(9.196\rho \right)$$

(25) $$c\left(\rho \right)=26.73-320.2\rho +1640\rho^{2}-4090\rho^{3}+4991\rho^{4}-2366\rho^{5}$$

(26) $$d\left(\rho \right)=2.64-40.06\rho +256\rho^{2}-765.7\rho^{3}+1150\rho^{4}-668.8\rho^{5}$$

(27) $$\begin{array}{c} m\left(\rho \right)=2449.63672\rho -9036.53466\rho^{2}+14751.60684\rho^{3}\\ -9014.68531\rho^{4}-279.06486 \end{array}$$

(28) $$n\left(\rho \right)=1.717+0.1722\cos\left(7.059\rho \right)+0.2572\sin\left(7.059\rho \right)$$

**Figure 20:**
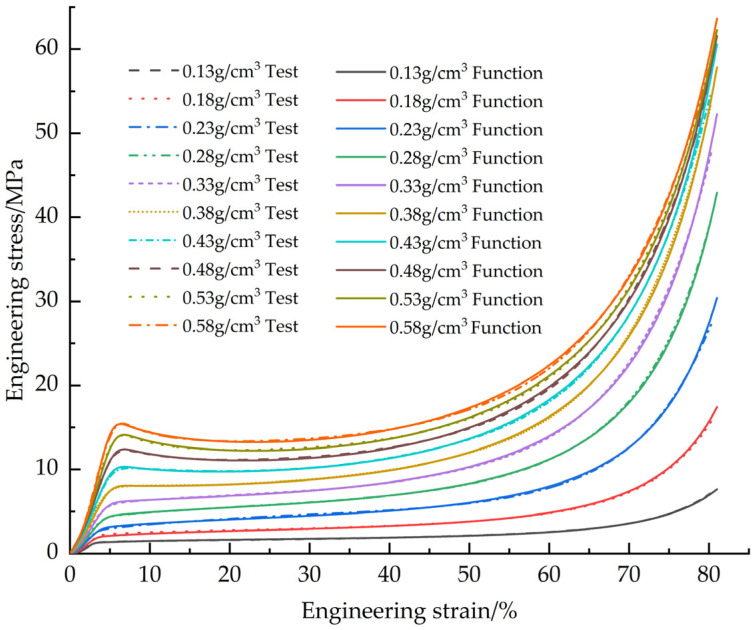
Corrected EPS compression constitutive model curves vs. test curves.

Figure 20 demonstrates the corrected EPS compression constitutive model fitting curves versus the test curves

**Figure 21:**
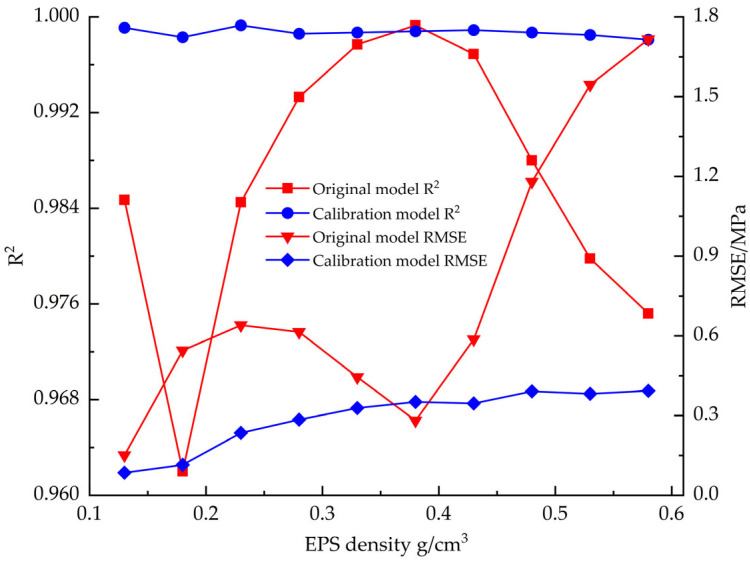
R^2^ vs. RMSE before and after EPS compression constitutive model correction.

Figure 21 compares the fitting accuracy parameters (R^2^ and RMSE) of the EPS compression constitutive model before and after correction, and the results show that the fit of the corrected model to the test data is significantly improved. The mean value of R^2^ is improved from 0.9861 to 0.9987, and the mean value of RMSE is reduced from 0.7704 to 0.2911.

**Figure 22:**
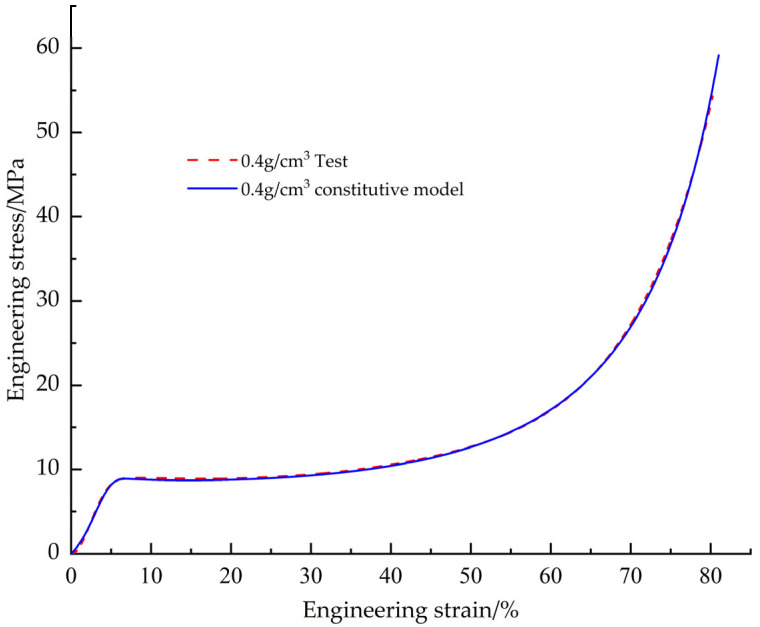
Corrected EPS compression constitutive model curve vs. test curve.

Similarly, in order to verify the accuracy of the corrected EPS compression principal model, the EPS compression test data with a density of 0.4 g/cm^3^ were also utilized for comparison (Figure 22). The goodness of fit between the engineering stress-strain curve obtained from the test and the predicted curve of the constitutive model was calculated to be 0.9996, and the root-mean-square error was 0.1972, which shows that the corrected EPS compression constitutive model is able to accurately predict the mechanical response of the EPS material under compression conditions.

## 5. Finite Element Simulation

In the simulation of this paper, the part size unit is millimeter (mm), and the stress cloud diagram legend unit is megapascal (MPa).

### 5.1. Finite Element Validation of the EPS Constitutive Model

Although ABAQUS provides a rich library of materials, in order to more accurately characterize the mechanical properties of the homemade materials, it is necessary to use the constructed material constitutive model, write a material subroutine using the Fortran language, and apply the properties through the subroutine interface provided by ABAQUS. In view of the fact that the nozzle cover is subjected to transient shock loads during the engine ignition to opening process, the VUMAT subroutine suitable for explicit dynamics analysis was written.

The EPS tensile and shear constitutive models were validated using the VUMAT interface provided by ABAQUS, and the EPS compression constitutive model was validated using the crushable foam model that comes with ABAQUS.

The EPS specimens were prepared by mold foaming, so they were considered isotropic materials. The generalized Hooke’s law is utilized to extend the one-dimensional constitutive equations to three dimensions.

(29) $$\sigma_{ij}=\lambda \varepsilon_{kk}\delta_{ij}+2\mu \varepsilon_{ij}$$

In the formula: $\delta_{ij}$ is equal to 1 when i=j and 0 otherwise; λ and μ are Lamé constants.

(30) $$\lambda =\frac{E\upsilon }{\left(1+\upsilon \right)\left(1-2\upsilon \right)}$$

(31) $$\mu =\frac{E}{2\left(1+\upsilon \right)}$$

In order to accurately reproduce the real test, 1:1 solid modeling was used in this study, and the loading conditions consistent with the test were maintained. In view of the wide range of densities involved in the tests and the large amount of test data, the EPS material with an intermediate density of 0.43 g/cm^3^ was selected for modeling and analysis, and Poisson’s ratio was referenced to that of high-density polyethylene, which is 0.33 [23]. The material failure criterion is defined in the VUMAT subroutine through the maximum equivalent force criterion, which is equivalent to the MAPS damage model built into ABAQUS.

**Figure 23:**
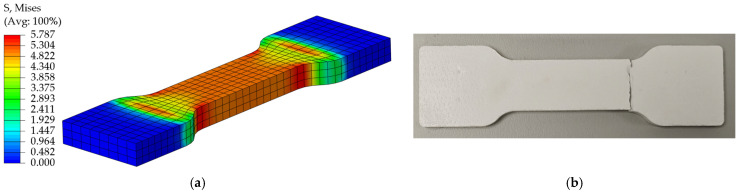
(**a**) Simulated stress cloud of EPS tensile specimen; (**b**) Fracture diagram of EPS tensile specimen test.

**Figure 24:**
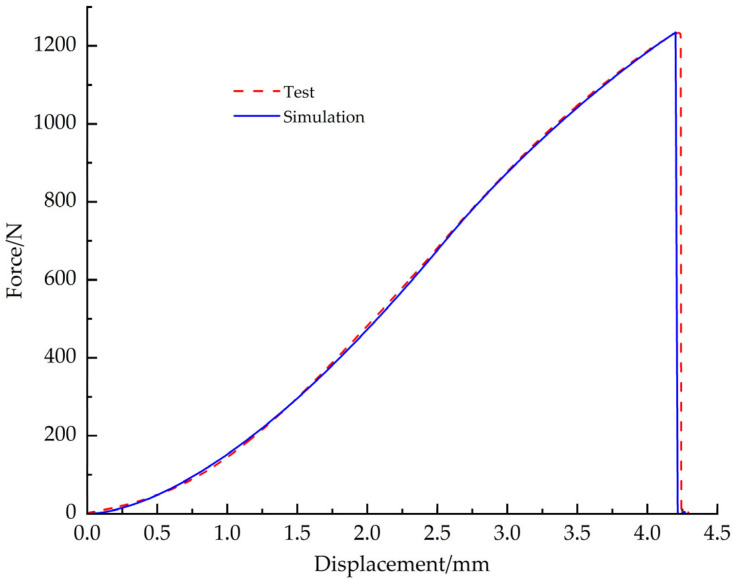
Tensile simulation curve vs. test curve.

**Figure 25:**
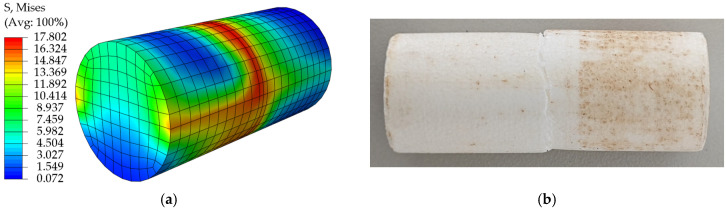
(**a**) Simulated stress cloud of EPS shear specimen; (**b**) Fracture diagram of EPS shear specimen test.

**Figure 26:**
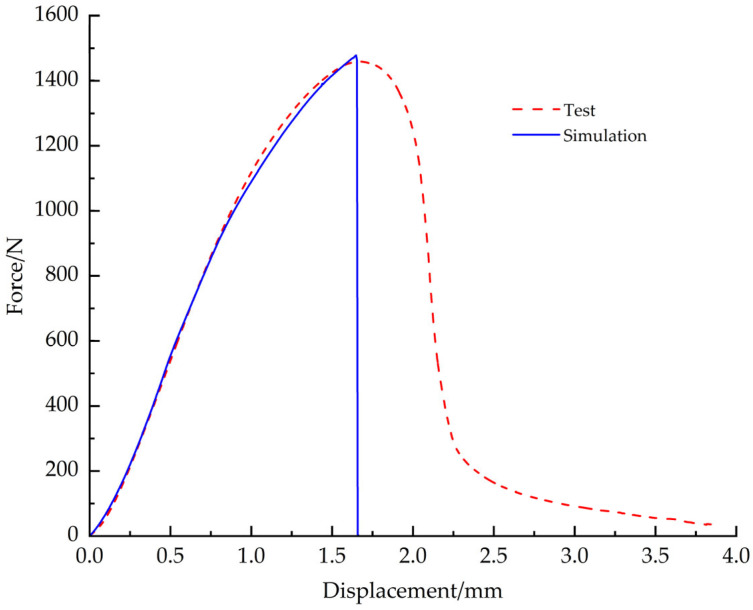
Shear simulation curve vs. test curve.

Comparing the simulation results with the test data, the load-displacement curves are in good agreement. Under the tensile condition, the simulation cloud and the real fracture shape are shown in Figure 23a and Figure 23b, respectively, and the load-displacement curves of simulation and test are shown in Figure 24; under the shear condition, the simulation cloud and the real fracture shape are shown in Figure 25a,b, and the load-displacement curves for simulation and test are shown in Figure 26. The structural limitations of the test fixture cause the load-displacement curve of the test to exhibit a slow downward trend after exceeding the peak load (failure point). The fundamental reason is that, during the test, after the EPS material fails, the two sides of the fracture surface are forced into close contact and sliding under the action of the fixture, generating significant friction; as the fixture continues to displace, this friction gradually decreases; until the fracture surfaces ultimately separate completely, and the friction disappears. Simulation analysis primarily considers the mechanical behavior prior to failure and uses the maximum stress criterion to determine material failure. Therefore, the simulation load-displacement curve exhibits brittle fracture characteristics similar to those observed in tensile tests after reaching the failure point.

To address the mesh distortion problem in the large deformation compression simulation, the built-in material model of ABAQUS is used in this study to validate the EPS constitutive relationship. In order to avoid the lateral mesh protrusion caused by large deformation, upper and lower rigid platens are set in the simulation model to simulate the real compression test conditions.

**Figure 27:**
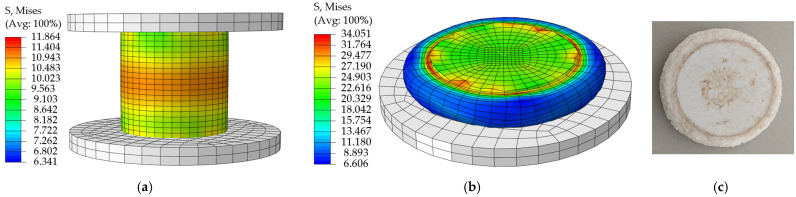
(**a**) Stress cloud at the beginning of EPS compression specimen simulation; (**b**) Stress cloud at the end of simulation of EPS compression specimen; (**c**) EPS compression specimen after compression.

**Figure 28:**
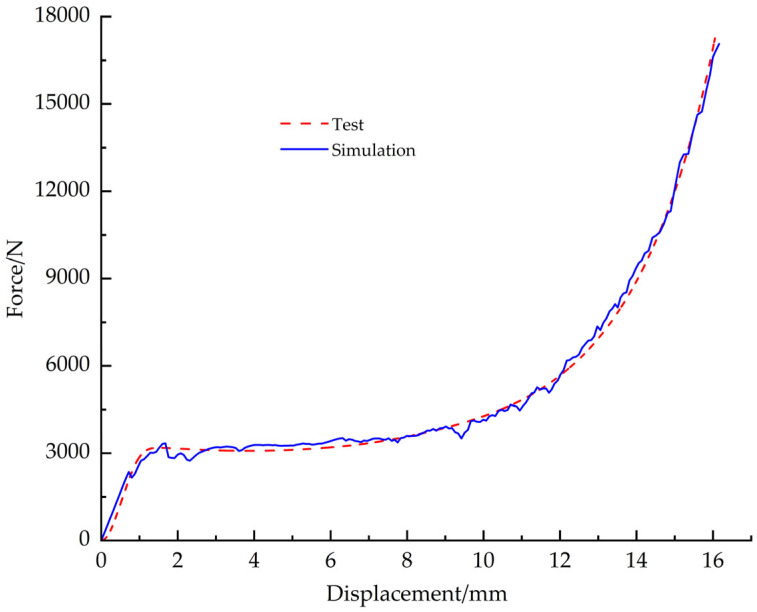
Compression simulation curve vs. test curve.

The material model parameters are shown in Table 8. The compressive yield stress ratio of 0.75 measured by Han S.H. et al. [24] for polyurethane foam was used, and the foam hardening parameters were based on the true stress-strain data. No failure criterion was defined for the EPS compression material model since no rupture of the specimen occurred at the end of the compression test. The simulation start and end cloud plots for the compression condition are shown in Figure 27a and Figure 27b, respectively; the test results are plotted in Figure 27c, and the load-displacement curves for simulation and test are shown in Figure 28.

### 5.2. Finite Element Simulation of EPS Nozzle Cover

#### 5.2.1. Simulation Model and Nozzle Cover Material Model Allocation Scheme

In this study, numerical simulation using the display dynamics method is used to analyze the pressure-bearing performance of the EPS nozzle cover with a density of 0.43 g/cm^3^.

In order to simplify the model, the nozzle is regarded as a rigid body, and its mechanical response is ignored in the simulation, and the failure of the adhesive layer is not considered. The opening pressure of the nozzle cover is mainly determined by the mechanical properties and structural design of the material. Based on the pure shear theory of a thin-walled circular plate, the formula for calculating the critical opening pressure of a thin-walled circular plate nozzle cover with uniform thickness is established as follows [5]:

(32) $$P=\left(4\tau h\right)/D$$

In the formula: P is the opening pressure; τ is the shear strength of the material; h is the thickness of the pressure surface; D is the diameter of the shear circle at the end face of the cover.

**Figure 29:**
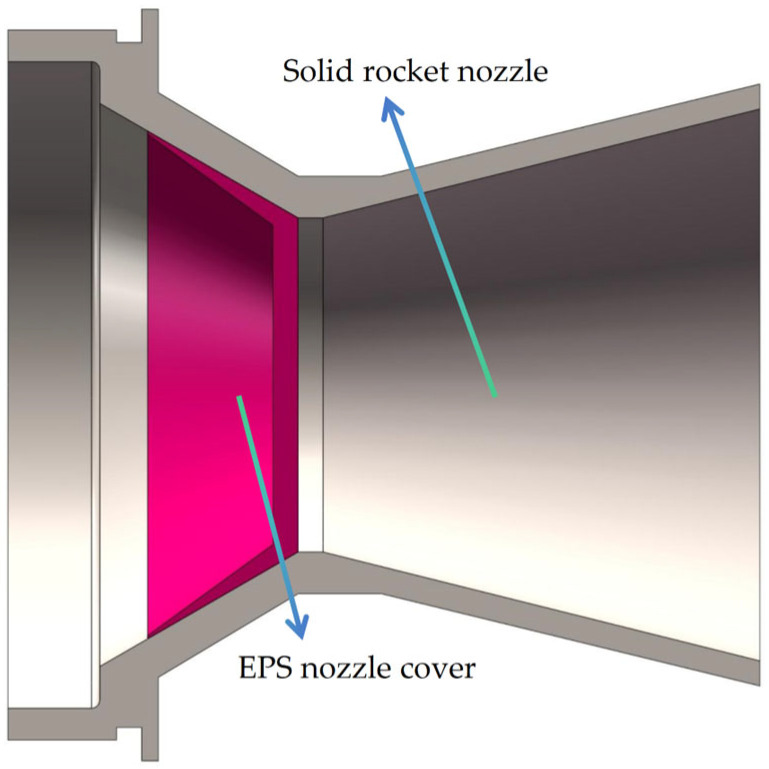
Cutaway view of the simulation model of the solid rocket motor nozzle and cover.

In this study, three different thicknesses of EPS nozzle covers (3 mm, 6 mm, and 9 mm) with the same effective pressure-bearing diameters at the ends (38.2 mm at the inner end face and 40 mm at the outer end face) were investigated. Numerical simulations were performed to determine the pressure-bearing capacity under unidirectional internal and unidirectional external pressure. Figure 29 shows the numerical simulation model of the solid rocket motor nozzle and cover.

**Figure 30:**
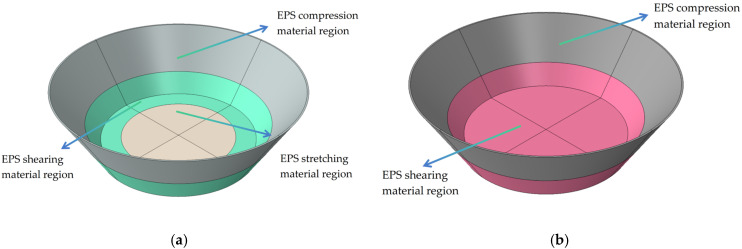
(**a**) Distribution of materials for nozzle cover for scheme 1; (**b**) Distribution of materials for nozzle cover for scheme 2.

Based on the stress distribution in the pressure-bearing process of the nozzle cover, two material partition design schemes are proposed in this study:

Scheme 1: the upper part of the sealing arc of the nozzle cover is an EPS compression material model, the center of the end face of the cover is a tensile EPS material model, and the part of the end face of the nozzle cover that is connected with the sealing arc of the cover is an EPS shear material model, and Figure 30a shows the distribution parts of the material model.

Scheme 2: The upper part of the sealing arc of the nozzle cover is modeled as EPS compression material, and the lower part of the sealing arc of the nozzle cover and the whole end face part are modeled as EPS shear material. Figure 30b shows the assigned parts of the material model.

The model mesh is divided by a structural hexahedron element (C3D8R). After the grid independence verification, the number of grid elements used for EPS nozzle cover simulation is 13,032 (3 mm), 17,670 (6 mm) and 25,956 (9 mm), respectively. The material model of the nozzle cover is selected from the VUMAT subroutine verified in the previous section, and the crushable foam model from ABAQUS. The remaining boundary conditions are set as follows:

- Analysis step setting: Create an explicit dynamic analysis step, define the analysis step time to be 0.1 s, and the remaining parameters remain the default setting.
- Loading method: The simulation is designed to predict the pressure-bearing capacity of the EPS nozzle cover, so a linearly increasing pressure load is applied to the nozzle cover, which is uniformly loaded from 0 MPa to 10 MPa in 0.1 s.
- Constraints: The rigid nozzle is assigned a fixed constraint. A tie constraint is applied to the bonded region between the EPS nozzle cover and the nozzle convergent section.

**Figure 31:**
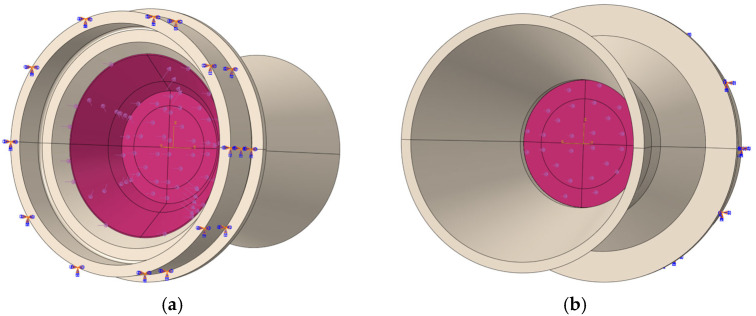
(**a**) Internal pressure loading and boundary diagram of nozzle cover; (**b**) External pressure loading and boundary diagram of nozzle cover.

The load zone and boundary conditions of the solid rocket motor nozzle and EPS nozzle cover simulation model are shown in Figure 31, where the symbol consisting of three arrows indicates a fully fixed constraint, and the purple single arrow symbol indicates the direction of pressure loading.

**Figure 32:**
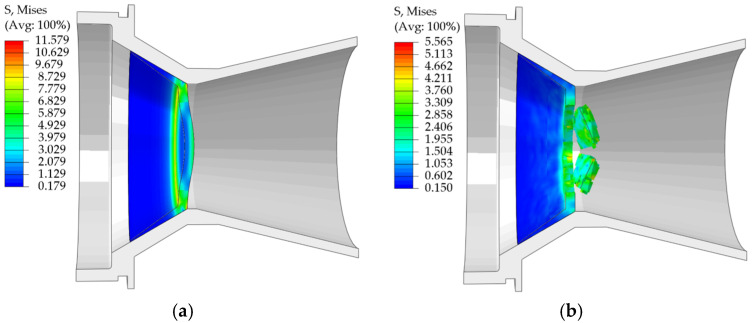
(**a**) Maximum stress cloud of nozzle cover for scheme 1; (**b**) Stress cloud of nozzle cover rupture for scheme 1.

With the material distribution of scheme 1, the simulation results showed that the maximum stress that the nozzle cover with a thickness of 3 mm could withstand was 11.579 MPa (Figure 32a). Under this scenario, the nozzle cover ruptures because the stress reaches the stress threshold of the EPS tensile material model, but the failure cross-section (Figure 32b) is not consistent with the actual test: the damage usually occurs at the connection between the sealing arc and the end face of the nozzle cover in the test, or it is shown as the center of the pressure-bearing end face cracking in the direction of the surroundings, while the simulation results fail to accurately reproduce this failure mode. Therefore, all subsequent simulation analyses were performed using the material allocation scheme of scheme 2.

#### 5.2.2. Simulation Results and Analysis

**Figure 33:**
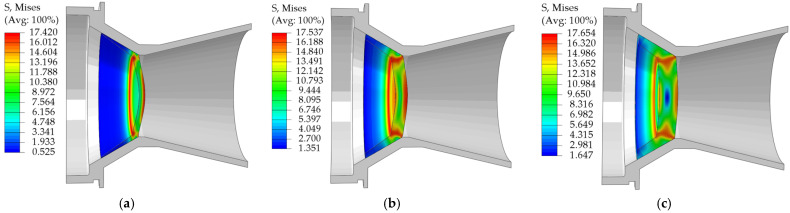
Maximum pressure stress cloud of EPS nozzle covers of different thicknesses under internal pressure conditions: (**a**) 3 mm; (**b**) 6 mm; (**c**) 9 mm.

Figure 33 illustrates the results of the numerical simulation of the unidirectional internal pressure nozzle cover pressurization.

**Figure 34:**
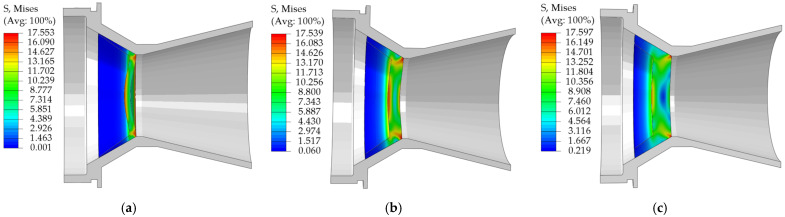
Maximum pressure stress cloud of EPS nozzle covers of different thicknesses under external pressure conditions:(**a**) 3 mm; (**b**) 6 mm; (**c**) 9 mm.

Figure 34 illustrates the results of the numerical simulation of the unidirectional external pressure nozzle cover pressurization.

Comparison of simulation and theoretical calculations of pressure capacity shows that when the thickness of the EPS nozzle cover is greater than 3 mm, the error in the pressure capacity increases significantly from the calculations based on the pure shear theory of thin-walled circular plates. To verify this phenomenon, further simulations were performed for 2 mm and 4 mm thickness nozzle covers close to 3 mm. The results show that the deviation between the simulated and theoretical values of the compressive limit under the internal pressure condition is 18.97% for the 4 mm-thickness cover, and the deviation under the external pressure condition is even higher at 26.88%, while for the 2 mm-thickness cover, the deviations under the internal and external pressure conditions are −11% and −8.6%, respectively.

The above data confirms that the pure shear theory for thin-walled circular plates is no longer applicable when the thickness of the EPS nozzle cover ends exceeds 3 mm. Table 9 details the simulated maximum pressure results for the EPS nozzle cover, the results of the theoretical equation and the error between the two.

Numerical simulation results show that the pressure characteristics of the EPS nozzle cover under unidirectional internal pressure and external pressure show a consistent pattern:

- The ability to withstand pressure increases with the thickness of the nozzle cover end.
- The stress is mainly concentrated in the bottom periphery and the center region, and the sealing arc stress along the axial direction with the gradual decay away from the bottom of the bearer.

This stress distribution characteristic matches well with the theoretical prediction, which effectively verifies the rationality of the material distribution scheme of Scheme 2.

Under unidirectional internal pressure, the stress field at the end face of the nozzle cover shows typical phase expansion characteristics: the stress at the outer end face firstly expands inwardly, and then the stress at the inner end face gradually strengthens and expands outwardly, and finally a significant stress concentration is formed around the periphery of the end face and the center region, which leads to the damage of the structure at the maximum equivalent stress.

Under unidirectional external pressure, although the stress field of the nozzle cover maintains a similar phase expansion pattern, the center region shows unique stress distribution characteristics. As in the case of internal pressure, the stresses at the outer end face expand inward first, and the stresses at the inner end face then spread outward. However, the center region shows obvious reverse characteristics—the inner end face stress is always higher than the outer end face stress, and with the increase of pressure, the stress field shows the evolution trend of radial expansion from the center to the outer end face and the surrounding area.

For the bowl-type EPS nozzle cover, when the wall thickness exceeds 3 mm, the internal pressure bearing performance is obviously better than the external pressure resistance under the same thickness.

## 6. Conclusions

In this paper, the mechanical properties of EPS specimens with different densities were tested in tensile, compression and shear, and their data were used to fit the constitutive model of EPS, and the constitutive model was corrected. Then, using the VUMAT subroutine interface of ABAQUS finite element simulation software and the material model that comes with the ABAQUS finite element simulation software, after verifying the accuracy of the material model, the effects of the thickness and pressure direction of the nozzle cover on the stress distribution and the pressure-bearing capacity were studied, and the following conclusions were obtained:

- The mechanical properties of EPS materials show obvious density-dependent laws: in the elastic deformation stage, their Young’s modulus and yield strength are positively correlated with the material density; in terms of the destructive properties, the tensile and shear fracture strains of the materials are significantly reduced with the increase of the density; in the process of compressive deformation, with the increase of the density, the plateau stage of the stress-strain curves is significantly shortened, and the densification transition occurs in advance. This systematic density effect reflects the evolutionary characteristics of the microstructure of EPS materials with density.
- In order to accurately characterize the mechanical behavior of EPS materials, the accuracy of the constitutive models was significantly improved in this study by introducing a correction term. The tensile and shear constitutive models constructed based on the Sherwood-Frost theoretical framework both use similar correction terms, while the compressive constitutive model is corrected by optimizing the Avalle model. The results of model correction showed that the fitting accuracy of each of the constitutive models was significantly improved: the R^2^ of the tensile constitutive model is increased from 0.9955 to 0.9996 (by 0.41%), and the RMSE is decreased from 0.1051 to 0.0276 (by 73.74%); the R^2^ of the shear constitutive model is increased from 0.9928 to 0.9996 (by 0.68%), and the RMSE is increased from 0.1288 to 0.9996 (by 0.68%), and the RMSE is increased from 0.9928 to 0.9996 (by 0.68%). RMSE decreased from 0.1288 to 0.01925 (85.05% reduction); R^2^ of the compression constitutive model increased from 0.9861 to 0.9987 (1.28% enhancement), and RMSE decreased from 0.7704 to 0.2911 (62.21% reduction). These data not only quantitatively demonstrate the correction effect but also confirm the effectiveness of the optimization method of the constitutive model.
- Through the comparative analysis of different material modeling schemes, the results show that the EPS nozzle cover design scheme using the compression-shear dual-material model can more accurately simulate the mechanical behavior under actual working conditions.
- As the thickness of the pressure-bearing surface at the end of the EPS nozzle cover increases, the error between the theoretical opening pressure and the simulation results increases significantly (reaching as high as 73.06% at a thickness of 9 mm). A comparison of the theoretical and simulation values at thicknesses of 2 mm and 4 mm shows that when the thickness exceeds 3 mm, the nozzle covers opening pressure formula based on the pure shear theory of thin-walled circular plates is no longer applicable.
- The pressure simulation results for EPS nozzle covers indicate that they exhibit excellent pressure-bearing capacity and performance, with 9 mm thick EPS nozzle cover capable of withstanding 7.58 MPa (under internal pressure conditions); its opening pressure is positively correlated with thickness, and under the same thickness, the internal pressure bearing capacity is higher than the external pressure, and as the thickness increases, this difference significantly expands—when the thickness reaches 9 mm, the internal pressure bearing capacity is 3.13 MPa higher than the external pressure condition.
- The results of this research can provide references for other engineering applications, including: proposing the form of correction term to improve the fit of the constitutive model; assigning the corresponding properties to the material according to the actual stress of the specimen during the finite element analysis to improve the accuracy of the simulation results; and providing theoretical support for the optimization of the structure and design of the performance of EPS products by using the experimental results and the corrected constitutive model of the EPS.

## Figures and Tables

**Table 1:** Material properties from the tensile test.

EPS Density (g/cm^3^)	Young’s Modulus/MPa	Maximum Stress/MPa	Strain at Failure/%
0.13	12.4242	1.4624	11.924
0.18	19.4137	2.0248	10.514
0.23	29.1715	2.6760	9.728
0.28	36.9152	3.0856	9.190
0.33	48.2400	3.7424	8.902
0.38	58.5971	4.3608	8.574
0.43	67.8006	4.9344	8.428
0.48	77.3844	5.4232	8.158
0.53	97.0053	6.3080	7.822
0.58	114.9935	7.3048	7.566

**Table 2:** Material properties from the shear test.

EPS Density (g/cm^3^)	Young’s Modulus/MPa	Maximum Stress/MPa	Strain at Failure/%
0.13	8.5148	1.0177	22.760
0.18	14.3804	1.6067	17.845
0.23	20.5846	2.2858	15.755
0.28	28.8131	2.8111	12.895
0.33	30.1682	3.3927	10.760
0.38	53.5426	4.0315	9.230
0.43	68.6947	4.6443	8.345
0.48	85.2817	5.3210	7.860
0.53	105.0286	6.2969	7.450
0.58	131.3217	7.6358	7.180

**Table 3:** Material properties from the compressive test.

EPS Density (g/cm^3^)	Young’s Modulus/MPa	Yield Stress/MPa	Yield Strain/%
0.13	51.0378	1.3570	3.725
0.18	80.8732	2.1957	4.050
0.23	110.5114	2.8290	4.190
0.28	142.6201	4.2223	4.580
0.33	172.3353	5.7689	5.205
0.38	209.5474	7.8104	5.775
0.43	245.9460	9.9753	6.175
0.48	273.8016	12.1913	6.470
0.53	305.7203	14.0444	6.580
0.58	333.5680	15.4311	6.340

**Table 4:** Parameter values of the shape function in the EPS tensile constitutive model.

Parameter	Value
A_1_	0.1173
A_2_	0.004029
A_3_	−0.002634
A_4_	0.002145
A_5_	−0.0005482
A_6_	6.155 × 10^−5^
A_7_	−3.23 × 10^−6^
A_8_	6.497 × 10^−8^

**Table 5:** Density item unknown parameter value.

EPS Density (g/cm^3^)	Parameter A	Parameter B
0.18	0.5116	0.0631
0.23	0.7073	0.04763
0.28	0.7572	0.03489
0.33	0.7504	0.03516
0.38	0.7682	0.03205
0.43	0.8462	0.02469
0.48	0.9308	0.01849
0.53	1.02	0.01582
0.58	1.091	0.01343

**Table 6:** Parameter values of the shape function in the EPS shear constitutive model.

Parameter	Value
A_1_	0.1339
A_2_	−0.003972
A_3_	−0.0003814
A_4_	3.11 × 10^−5^
A_5_	−6.27 × 10^−7^

**Table 7:** Values of the parameters m and n.

Density (g/cm^3^)	Parameter m	Parameter n
0.13	−12.93	1.338
0.18	−11.79	1.17
0.23	−7.984	1.372
0.28	−9.99	1.541
0.33	−9.058	1.638
0.38	−9.959	1.719
0.43	−8.859	1.763
0.48	1.353	1.865
0.53	1.426	1.958
0.58	1.309	2.005

**Table 8:** Crushable foam material properties.

Density (t/mm^3^)	Young’s Modulus/MPa	Compression Yield Stress Ratio	Poisson’s Ratio
4.3 × 10^−10^	260.58	0.75	0.33

**Table 9:** Maximum pressure of the EPS nozzle cover.

Thickness/mm	Internal/MPa	Formula/MPa	Relative Error/%	External/MPa	Formula/MPa	Relative Error/%
2	0.86	0.97	−11.34	0.85	0.93	−8.60
3	1.49	1.46	2.05	1.29	1.39	−7.19
4	2.32	1.95	18.97	2.36	1.86	26.88
6	4.35	2.92	48.97	3.67	2.79	31.54
9	7.58	4.38	73.06	5.45	4.18	30.38

## Data Availability

The original contributions presented in this study are included in the article. Further inquiries can be directed to the corresponding author.
